# Pathogenicity of Human ST23 *Streptococcus agalactiae* to Fish and Genomic Comparison of Pathogenic and Non-pathogenic Isolates

**DOI:** 10.3389/fmicb.2017.01933

**Published:** 2017-10-06

**Authors:** Rui Wang, Liping Li, Yin Huang, Ting Huang, Jiayou Tang, Ting Xie, Aiying Lei, Fuguang Luo, Jian Li, Yan Huang, Yunliang Shi, Dongying Wang, Ming Chen, Qiang Mi, Weiyi Huang

**Affiliations:** ^1^Guangxi Key Laboratory for Aquatic Genetic Breeding and Healthy Aquaculture, Guangxi Institute of Fisheries, Nanning, China; ^2^Institute of Animal Science and Technology, Guangxi University, Nanning, China; ^3^Hechi Center for Animal Disease Control and Prevention, Hechi, China; ^4^Aquatic Animal Disease Pevention and Control Laboratory, Liuzhou's Aquaculture Technology Extending Station, Liuzhou, China; ^5^School of Life Sciences, Fudan University, Shanghai, China; ^6^Guangxi Center for Disease Control and Prevention, Nanning, China; ^7^Aquaculture Laboratory, Guangxi Aquaculture and Animal Husbandry School, Nanning, China

**Keywords:** *Streptococcus agalactiae*, Group B *Streptococcus* (GBS), pathogenicity, genomic comparison, sequence type (ST), ST23

## Abstract

*Streptococcus agalactiae*, or Group B *Streptococcus* (GBS), is a major pathogen causing neonatal sepsis and meningitis, bovine mastitis, and fish meningoencephalitis. CC23, including its namesake ST23, is not only the predominant GBS strain derived from human and cattle, but also can infect a variety of homeothermic and poikilothermic species. However, it has never been characterized in fish. This study aimed to determine the pathogenicity of ST23 GBS to fish and explore the mechanisms causing the difference in the pathogenicity of ST23 GBS based on the genome analysis. Infection of tilapia with 10 human-derived ST23 GBS isolates caused tissue damage and the distribution of pathogens within tissues. The mortality rate of infection was ranged from 76 to 100%, and it was shown that the mortality rate caused by only three human isolates had statistically significant difference compared with fish-derived ST7 strain (*P* < 0.05), whereas the mortality caused by other seven human isolates did not show significant difference compared with fish-derived ST7 strain. The genome comparison and prophage analysis showed that the major genome difference between virulent and non-virulent ST23 GBS was attributed to the different prophage sequences. The prophage in the P1 region contained about 43% GC and encoded 28–39 proteins, which can mediate the acquisition of YafQ/DinJ structure for GBS by phage recombination. YafQ/DinJ belongs to one of the bacterial toxin–antitoxin (TA) systems and allows cells to cope with stress. The ST23 GBS strains carrying this prophage were not pathogenic to tilapia, but the strains without the prophage or carrying the pophage that had gene mutation or deletion, especially the deletion of YafQ/DinJ structure, were highly pathogenic to tilapia. In conclusion, human ST23 GBS is highly pathogenic to fish, which may be related to the phage recombination.

## Introduction

*Streptococcus agalactiae*, or Group B *Streptococcus* (GBS) is an important pathogen that causes sepsis and meningitis in newborns, mastitis in bovine, and meningoencephalitis in fish (Tazi et al., 2011; Lamagni et al., 2013). As commensal bacteria, GBS colonize the gastrointestinal and genitourinary tracts of 10–30% of healthy human population and can infect the elderly subjects with underlying conditions (Hansen et al., 2004; van der Mee-Marquet et al., 2008; Skoff et al., 2009). Multilocus sequence typing (MLST), the current reference method for GBS genotyping, is able to distinguish many sequence types (STs) based on alleles for seven housekeeping genes, which can subsequently be clustered into clonal complexes (CC) reflecting the phylogenetic structure of the GBS population (Jones et al., 2003; Feil et al., 2004). Defined phylogenetic lineages are associated with specific pathogenicity (Jones et al., 2003). Most of human carriage and clinical isolates are clustered into six major CCs, namely CC1, CC10, CC17, CC19, CC23, and CC26, and the majority of bovine isolates belong to the CC67/61, and the main GBS strains associated with fish are members of a limited number of CCs, namely CC7 and CC552 (Jones et al., 2003; Evans et al., 2008; Sorensen et al., 2010; Delannoy et al., 2013; Almeida et al., 2016; Jiang et al., 2016). However, it is notable that the specific pathogenicity may not be absolute. Most of lineages can infect multiple hosts including human, cattle, fish, et al. and there is an existence of cross-infectivity. Fish is an important source of protein nutrition for humans. It has been shown that consumption of fish is associated with an increased risk of GBS colonization in people (Foxman et al., 2007). In 2015, an outbreak of GBS infection was related to consuming infected raw freshwater fish, which affected more than 200 patients in Singapore (Tan et al., 2017). Thus, it is proposed that some strains of aquatic GBS may present a zoonotic or anthroponotic hazard and the fish may be a reservoir of pathogenic GBS. Therefore, it is necessary to investigate the pathogenicity of GBS CCs to fish. The infection studies have demonstrated that human CC7 and CC19, as well as cow CC103 may infect tilapia (Evans et al., 2009; Chen et al., 2015). CC23 is not only the predominant CC for human and cattle, but also can infect aquatic mammals and poikilotherms, such as seals and crocodiles (Delannoy et al., 2013; Godoy et al., 2013). However, it has never been characterized in fish, which could be due to lack of exposure rather than lack of virulence (Delannoy et al., 2016). The challenge experiments using ST23 GBS have shown that the human-derived, but not seal-derived GBS can infect tilapia (Chu et al., 2016; Delannoy et al., 2016). More challenge experiments are definitely needed to evaluate the difference of pathogenicity between different hosts-derived ST23 GBS isolates, while the characterization of mechanism underlying the different pathogenicity is also important for the risk assessment of GBS-caused cross-host infection.

Comparative genome analysis between bacterial strains with different host specificity or virulence may help to rapidly screen the dispensable genes, gene deletions or mutations, and proteins that are differentially expressed; it is also an effective way to study the mechanisms of GBS cross-host infection, pathogenicity and immunogenicity (Evans et al., 2008; Rosinski-Chupin et al., 2013). Comparative genomic studies indicated that strains belonging to the ST260-261 types, which are specialized to the fish host, may have been divergent by reduction evolution over a long time, and the fish CC7 strains cannot be distinguished from human CC7 counterparts (Liu et al., 2013; Rosinski-Chupin et al., 2013). Phylogenomic analysis of 161 ST283 isolates from humans and fish revealed that these isolates formed a tight clade distinguished by 93 single-nucleotide polymorphisms (Kalimuddin et al., 2017). Although there is a high degree of similarity in genome sequence of strains within same CC, the differences still exist, which result in differences in traits between strains. Recombination occurs frequently and can involve a large area of the GBS genome, which is a major driver of GBS genetic diversity (Luan et al., 2005; Lefebure and Stanhope, 2007; Brochet et al., 2008b; Da Cunha et al., 2014). Analysis of sequenced GBS strains representing whole species diversity revealed a mosaic organization (Tettelin et al., 2005). Integrative and conjugative elements (ICEs) and prophages can also cause genome diversity and the emergence of virulent clones within the species through horizontal gene transfer (Brochet et al., 2008a). In fact, the prophage DNA content that accounts for up to 10% of the dispensable genome is specific to each intraspecies lineage, which highlights the key role for lysogeny on the evolution of bacterial inheritance (Domelier et al., 2009; Salloum et al., 2011). It has been shown that ST283 can not only colonize and infect various farmed freshwater fish, but also causes unusually severe invasive disease in humans, its pathogenicity may be related to prophage recombination (Kalimuddin et al., 2017).

GBS spread quickly through the fish and water, and can cause a large area of outbreak in aquaculture within a short period. However, the use of aquaculture antibiotics was far from the norm in application in humans, which more easily leads to the emergence of GBS resistant strains. Therefore, the spread of human GBS in aquaculture will pose a serious threat to human health, thus more attention should be paid to the cross-host transmission of GBS. The aim of the study was to determine the pathogenicity of ST23 GBS to fish, as well as to investigate the mechanisms of pathogenicity differences by genomic comparison.

## Materials and methods

### Sources of bacteria

Ten invasive serotype Ia ST23 GBS isolates were studied, including isolates recovered from women and man who suffered from vaginitis, cervicitis or urethritis, etc in China. The positive control, fish-derived GBS strain HN016 was isolated from an outbreak epidemical disease in tilapia from China, which belonged to serotype Ia and ST7. Prior to the challenge experiment, the strains were passed through fish to enhance their virulence post-storage (Eldar et al., 1995). The strains were passed through three Nile tilapia (weight 31.81 ± 0.33 g) one time each by intraperitoneal (IP) injection of ~10^7^ CFU/fish. Specimens from a freshly dead fish or moribund fish after challenge were cultured on TSA, and GBS was recovered from the liver and brain. According to our previous report, the specific PCR was used to type the colony isolated from the brain as GBS (Chen et al., 2012), and used for experimental infection.

This study was carried out in accordance with the recommendations of the principles and procedures of the Ethical Committee for Animal Experiments of Guangxi Institute of Fisheries. The protocol was approved by the Ethical Committee for Animal Experiments of Guangxi Institute of Fisheries.

### The pathogenicity of human ST23 GBS isolates to tilapia

Non-infected Nile tilapia (*Oreochromis niloticus)*, with an average weight of 63.15 ± 2.23 g were provided by the National Tilapia Seed Farm (Nanning, Guangxi, China). Before the experiments, the fish were acclimated in the fiber-reinforced plastic tanks (800 L each) with stocking rate of 4 g/L at 30 ± 4°C for 2 weeks. Each experimental group was kept in a 40 L plastic tank equipped with an independent recirculation system with an external biological filter (Haisheng, China). The water temperature was 30 ± 4°C and the fish were fed two times a day with formula feed (Tongwei Feed Company, Nanning, China). Prior to bacterial challenge, experimental fish were examined to be negative for bacterial infection. Ten fish were selected randomly and their brain and liver were cultured on TSA with 5% sheep blood. The plates were incubated at 33°C for 72 h and absence of microbial growth was confirmed.

A single colony of each bacterial isolate that passaged in tilapia was inoculated in TSB medium and cultured at 33°C for 20 h with a shaking speed of 120 rpm. The bacterial density was measured by colony forming unit (CFU) per mL as described previously (Chen et al., 2015). Twenty tilapia were randomly divided into a group. Each fish was IP injected with about 1 × 10^7^ CFU bacteria in 0.5 mL suspension, and control fish were injected with the same volume of sterile PBS. At 12 h post-infection, the brain, liver, spleen, head kidney, and intestine of infected tilapia were collected from the freshly dead fish. For live fish group, the animals were sacrificed with high concentration of benzocaine (300 mg/L) before the tissues were collected. Following standard fixation in 10% neutral buffered formalin and sample processing in paraffin wax blocks, paraffin sections (6 μm thick) were stained with hematoxylin and eosin (H&E) for light microscopy observation. The infected fish were observed and fed two times a day for 15 days. At the end of the experiment, the brain and liver samples were collected from all dead and surviving fish and the bacteria were isolated and determined as described above. Each group had three replicates. The data were analyzed by one-way ANOVA program available in SPSS software (version 19.0). Differences were analyzed by Tukey's multiple pair wise comparison, those with *P* < 0.05 were considered statistically significant.

### Genome sequencing, assembly, and annotation

The genomes of the 10 ST23 GBS strains were determined using Illumina HiSeq2000 sequencing platforms, and they were assembled with the ABySS program (Simpson et al., 2009). The minimal coverage was 500-fold. The whole genome shotgun (WGS) sequences of the 10 ST23 GBS strains have been deposited in GenBank and the accession numbers were listed in Supplementary Table 1. The assembled sequences were uploaded to the RAST website for gene function annotation^1^ In addition, the genomes of 13 GBS strains were selected for evolution analysis. The strains involved in this study were listed in Supplementary Table 1. Their ST type, the serotype, host, GC content, geographical origin and GenBank accession number were indicated.

### Prophage analysis, CRISPR analysis, and phylogenetic reconstruction

PHASTER was used to identify prophage sequences with default parameters (Arndt et al., 2016). CRISPRs finder and CRISPR recognition tool V1.0 were used to identify clustered regularly interspaced short palindromic repeats (CRISPRs) with default parameters (Bland et al., 2007; Grissa et al., 2007). The result of CRISPR was analyzed and modified according Lier et al.'s findings (Lier et al., 2015). OrthoMCL was used to delineate orthologous protein sequences among the isolates (Li et al., 2003). Multiple sequence alignment of single copy homologous protein sequences was performed using MAFFT, and poorly aligned positions and divergent regions of the alignment were removed (Katoh and Standley, 2013). By using PhyML program in Protest, the maximum likelihood (ML) estimation of phylogenetic trees and model parameters were performed, and the optimal amino acid substitution model was obtained by comparing AIC and BIC scores (Darriba et al., 2011). The ML based phylogenetic tree was constructed via RaxML software with 1,000 bootstrap replications (Stamatakis, 2014). Visualization of the phylogenetic tree were conducted using FigTree 1.4.3^2^

### Genomic comparison and pan-genome analysis

Eleven genome sequences of GBS from human, seal, tilapia or bovin were annotated by Rapid Annotation using Subsystem Technology (RAST) automated web service (Overbeek et al., 2014). The shared and unique genes among strains of ST23 GBS were checked by the sequence based genomic comparison tool that was provided by SEED viewer (Overbeek et al., 2014). The genome sequences of NNA011 or MRI Z1-201 were used as reference genome, and the genome sequences from all other 10 strains were aligned to the reference genome. The result listed the genes of the reference organism in chromosomal order and displayed hits on the comparison organisms accordingly. Comparative genomic analysis was carried out from a list of selected genomes in Supplementary Table 1 by Roary with a blast identity cutoff of 97% (Page et al., 2015). Before the comparisons, for avoiding the possible deviations owing to the different annotation processes, the re-annotation of all the genomes were performed by Prokka version 1.12 (Seemann, 2014). Visualization of the pan-genome data was performed by Anvi'o (Eren et al., 2015).

## Results

### The pathogenicity of human ST23 GBS isolates to tilapia

**Figure 1:**
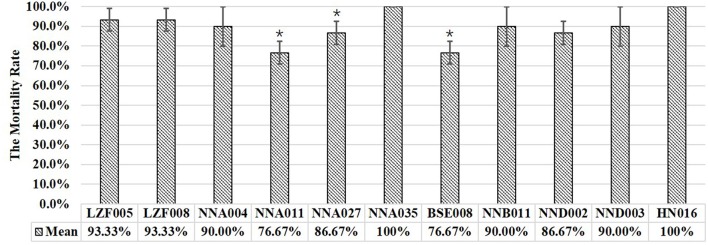
The results of experimental infection of tilapia with human ST23 GBS isolates. Mean represented the average cumulative mortality of three parallel groups. ^*^Indicated significant difference with HN016 infection (*P* < 0.05).

The results from the infection of tilapia with 10 isolates of human ST23 GBS were shown in Figure 1. The lowest mortality rate was 76.67% and the highest mortality rate was 100%. The mortality rate caused by isolates NNA011, NNA027, and BSE008 showed significant difference compared to the HN016 control group (*P* < 0.05), whereas the mortality rate caused by the other seven isolates did not show significant difference compared to the positive control group.

### Histopathological analysis tissue injury pathological analysis

**Figure 2:**
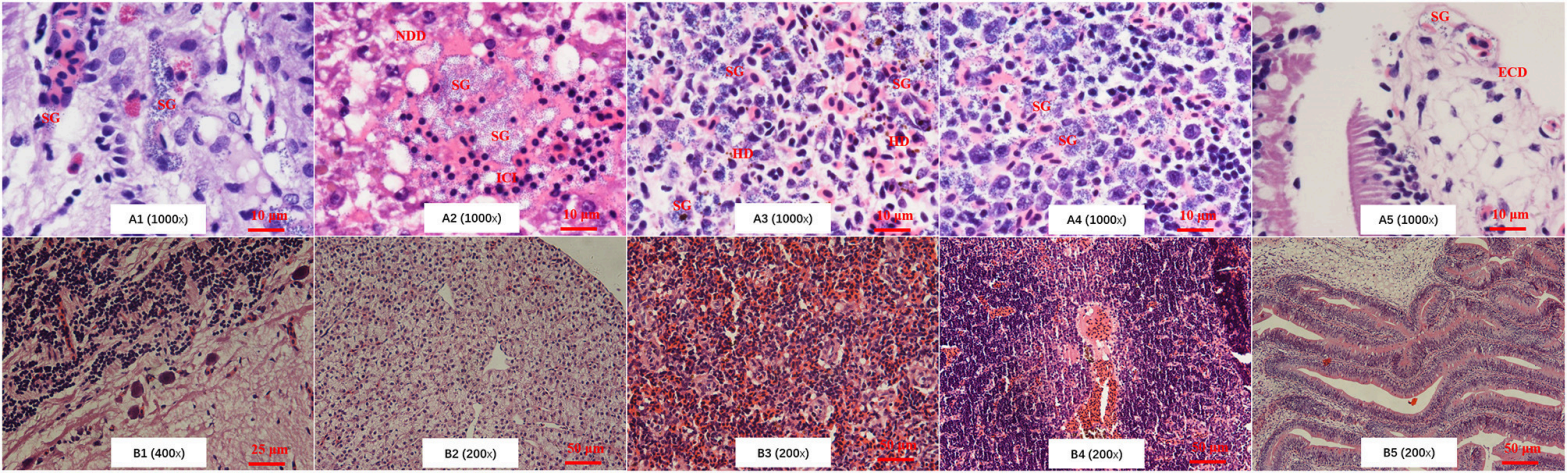
The histopathological changes of tilapia infected with GBS. **A1–A5** and **B1–B5** showed the histopathological results of brain, liver, spleen, kidney, and gut from tilapia injected with GBS or with PBS, respectively. There was no pathological damage in tissues from tilapia infected with PBS. SG, *Streptococcus* granules, NDD, Nucleus dissolve and disappear; ICI, Inflammatory cells infiltration; HD, hemosiderin deposition; ECD, epithelial cells desquamation.

Histopathological examination showed that there were severe lesions in the examined tissues of tilapia that infected by both GBS ST23 human-derived isolates and ST7 fish-derived strains (Figures 2A1–A5). For the brain tissue, the changes included edema, interstitial inflammatory cell infiltration, bleeding, large number of visible blue dye-stained *Streptococcus* particles (Figure 2A1). In the liver tissue, liver cells degeneration, necrosis, and disintegration, as well as the stained *Streptococcus* particles in the pancreas and the liver sinus were observed (Figure 2A2). The changes from spleen included serious disorder of tissue structure, red blood cell infiltration in white pulp area, and a large number of blue-stained *Streptococcus* granules in necrotic area (Figure 2A3). In addition, it was observed that head-kidney tissue structure was blurred and the number of lymphocytes decreased significantly, and there was a large number of necrotic lesions and stained *Streptococcus* granules in lesion area (Figure 2A4). Intestinal serosal boundary was blurred, and visible blue dye-stained *Streptococcus* granules were observed in serosa and submucosa (Figure 2A5). In contrast, fish injected with PBS did not show significant histopathological changes (Figures 2B1–B5). The histopathological results demonstrated the similar pathogenicity characteristics of ST23 GBS human isolates and ST7 GBS fish strains.

### Phylogenetic analysis

**Figure 3:**
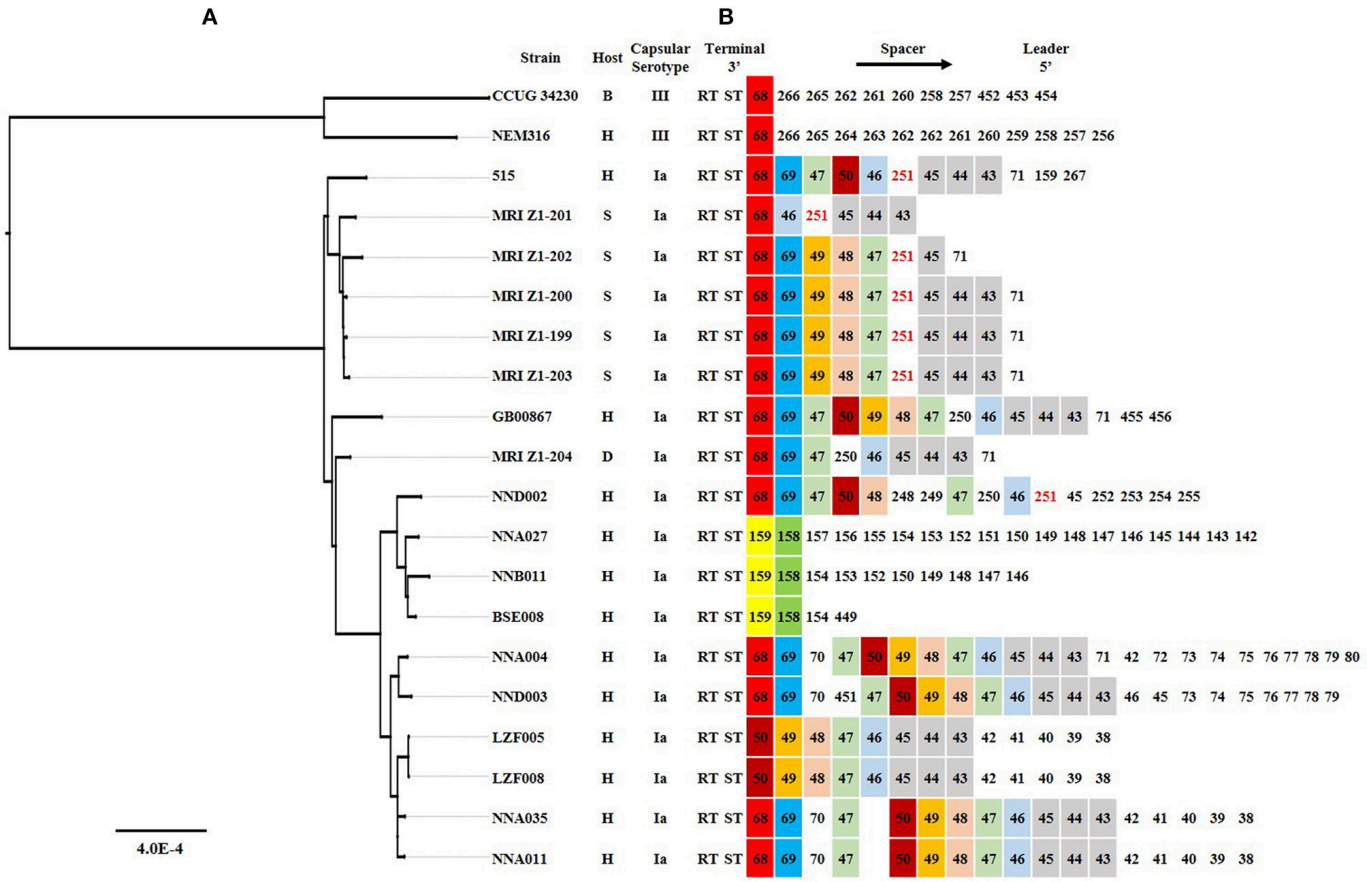
The phylogenetic analysis and CRISPR structure comparison for 20 different ST23 GBS strains. **(A)** A Maximum Likelihood phylogenetic tree based on 2,154 single copy orthology clusters of the 20 strains. **(B)** CRISPR loci comparison. Direct repeat sequence was not included; only RT, ST and spacers were represented. The spacer was numbered, and the same number highlighted with same color indicated that the spacer sequence was the same. H, S, B, and D indicated Human, Seal, Bovine, and Dog, respectively.

The phylogenetic relationships among 20 ST23 GBS strains from human, seal, bovine or dog were analyzed based on phylogenetic tree construction and CRISPR structure. The ML phylogenetic tree was construct (Figure 3) based on 469,453 amino acids of the 1,568 single copy orthology clusters from strains. It was shown that Ia and III were in different evolutional branches of ST23 GBS. The seal-derived ST23 GBS were in different evolutionary branches with the human-derived ST23 GBS that we used for tilapia challenge in this study. The CRISPR structure of ST23 GBS with different serotypes and hosts had the same terminal repeat (RT) and terminal spacer (ST) sequences, and the first spacer at three terminus from 75% (15/20) of strains was identical, whereas strains NNA027, BSE008 and NNB011 showed significantly different CRISPR structure from other Ia ST23 GBS.

### Prophage analysis

PHASTER revealed that there were 1–3 different prophages (intact, questionable or incomplete) in ST23 human- or seal-derived strains (Table 1). Some of these strains had prophages with 42.98–43.77% of GC, including six human-derived ST23 strains, five seal-derived ST23 strains, and one cow-derived ST61 strain. The total length of prophage shared by six human ST23 strains was 30.3 Kb, encoding 28 or 31 proteins. The length of prophage shared by five seal ST23 strains was 31.4 Kb or 34.5 Kb, encoding 35–39 proteins. The prophage length of cow ST61 strain was 31.2 Kb, encoding 32 proteins. The fish ST7 strain HN016 had a 46.2 kb length prophage with a GC content of 41.56%, which encoded 31 proteins.

**Table 1:** The analysis of prophage from GBS strains.

**Strain**	**Region**	**Region length**	**Total proteins**	Most common phage^*^	**GC(%)**
LZF005 (H/ST23)	1	12.9 Kb^inc^	14	Bacter_Diva_NC_028788(2)	30.85
LZF008 (H/ST23)	1	12.9 Kb^inc^	14	Bacter_Diva_NC_028788(2)	30.85
	2	11.9 Kb^inc^	6	Bacter_Diva_NC_028788(3)	38.13
	3	7.70 Kb^inc^	10	Salisa_1_NC_017983(1)	35.17
NNA004 (H/ST23)	1	54.1 Kb^int^	88	Strept_20617_NC_023503(13)	35.23
NNA011 (H/ST23)	1	30.3 Kb^que^	31	Clostr_phiCT453B_NC_029004(9)	43.71
NNA027 (H/ST23)	1	52.5 Kb^int^	79	Strept_20617_NC_023503(14)	35.51
	2	30.3 Kb^que^	31	Strept_phiARI0923_NC_030946(9)	43.76
NNA035 (H/ST23)	1	30.3 Kb^que^	31	Clostr_phiCT453B_NC_029004(9)	43.72
BSE008 (H/ST23)	1	46.6 Kb^int^	74	Strept_20617_NC_023503(14)	36.17
	2	49.6 Kb^int^	80	Strept_20617_NC_023503(14)	36.09
	3	30.3 Kb^que^	31	Clostr_phiCT453B_NC_029004(9)	43.77
NNB011 (H/ST23)	1	53.4 Kb^int^	82	Strept_20617_NC_023503(15)	35.41
	2	30.3 Kb^que^	31	Clostr_phiCT453B_NC_029004(9)	43.77
NND002 (H/ST23)	1	30.3 Kb^que^	28	Clostr_phiCT453B_NC_029004(9)	43.71
	2	45.0 Kb^int^	69	Strept_20617_NC_023503(13)	36.13
	3	48.1 Kb^int^	75	Strept_20617_NC_023503(13)	36.06
NND003 (H/ST23)	1	50.9 Kb^int^	82	Strept_20617_NC_023503(13)	35.23
	2	30.3 Kb^que^	31	Gordon_Nymphadora_NC_031061(9)	43.71
515 (H/ST23)	1	15.8 Kb^inc^	34	Strept_T12_NC_028700(10)	34.75
	2	29.7 Kb^int^	37	Strept_phi3396_NC_009018(9)	36.05
NEM316 (H/ST23)	1	10.4 Kb^inc^	10	Strept_9874_NC_031023(2)	36.78
GB00867 (H/ST23)	1	43.4 Kb^int^	57	Lactob_PLE3_NC_031125(20)	39.33
HN016 (F/ST7)	1	46.2 Kb^que^	31	Clostr_phiCT453B_NC_029004(9)	41.56
MRI Z1-199 (S/ST23)	1	49.3 Kb^int^	69	Lactob_PLE3_NC_031125(13)	36.80
	2	34.5 Kb^que^	38	Gordon_Kita_NC_031233(9)	43.36
MRI Z1-201 (S/ST23)	1	31.4 Kb^que^	35	Clostr_phiCT453B_NC_029004(9)	42.98
MRI Z1-200 (S/ST23)	1	34.5 Kb^que^	39	Gordon_Kita_NC_031233(9)	43.36
	2	59.7 Kb^int^	71	Strept_phiARI0131_2_NC_031941(13)	36.33
MRI Z1-202 (S/ST23)	1	34.5 Kb^que^	39	Gordon_Kita_NC_031233(9)	43.35
MRI Z1-203 (S/ST23)	1	34.5 Kb^que^	39	Gordon_Kita_NC_031233(9)	43.36
	2	59.7 Kb^int^	71	Strept_phiARI0131_2_NC_031941(13)	36.33
FSL S3-026 (B/ST67)	1	31.2 Kb^que^	32	Clostr_phiCT453B_NC_029004(9)	43.20
	2	48.5 Kb^que^	32	Strept_phiARI0746_NC_031907(10)	40.89
	3	15.0 Kb^inc^	22	Staphy_SPbeta_like_NC_029119(4)	35.62

*^int, que^ or ^inc^ represented the predicted completeness of prophage was intact, questionable or incomplete, respectively. H, S, B, and F indicated human, seal, bovine and fish, respectively*.

* *The phage(s) with the highest number of proteins most similar to those in the region*.

### Coding sequence alignment and analysis of aequence variation

**Figure 4:**
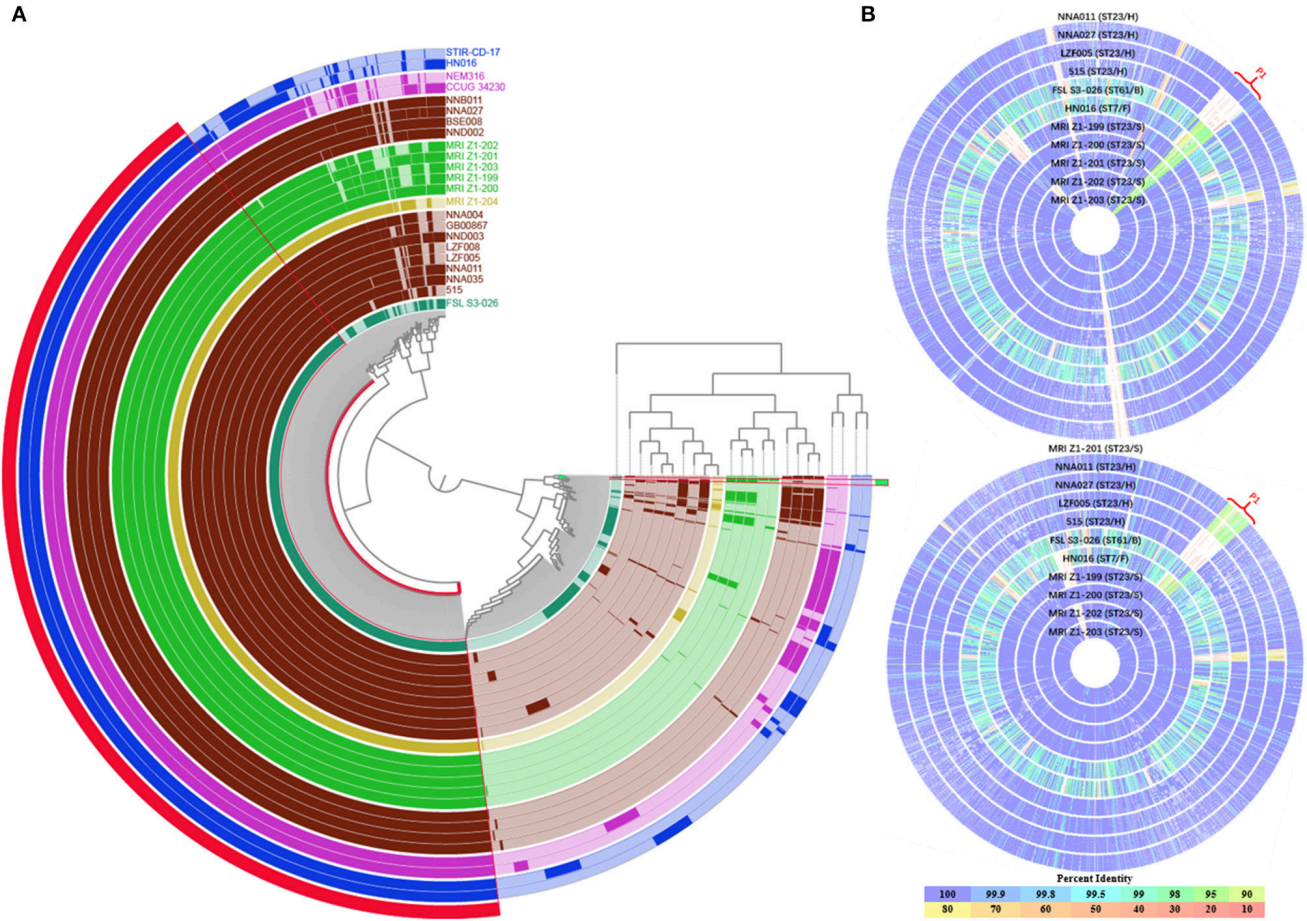
Comparison of GBS genomes. **(A)** Anvi'o pangenome visualization of 23 GBS genomes. The outer in red, showed the core genome of GBS, protein clusters shared among all the strains. The outer in green, showed the protein clusters shared among seal- and bovine-derived strains (P1 in **B)**. The genomes were clustered based on the presence/absence pattern of protein clusters. Human Ia ST23 strains were highlighted in brown, seal Ia ST23 strains were highlighted in green, fish strains were highlighted in blue, bovine strain FSL S3-026 was highlighted in turquoise. **(B)** Comparison of genomes of 11 GBS strains. The H021 genome and the MRI Z1-201 genome were served as the reference respectively, and other genomes were aligned; the alignment and the figure were created by the SEED Viewer; each circle represented a different genome, the strain name was labeled on each circle. The circle correlated to reference genome was not shown; the color bar on the bottom indicated the percentage of protein sequence identity against the reference genome. H, S, B, F, and D indicated human, seal, bovine, fish, and dog, respectively.

A comparative genomic analysis of human ST23 GBS was performed against 13 publicly available genome sequences from human, seal, bovine, fish and dog-derived strains (Supplementary Table 1). Pangenome analysis resulted in the identification of a set of 1,410 core genes (present in all the strains) and a set of 202 soft core genes (present in 95–99% of the strains). For the shell genes, 568 genes were identified between 15 and 95% of the strains, while 1,798 genes were present in <15% of the strains. Based on this analysis, we identified three genes from human ST23 were not present in any of the seal ST23 strains, which all encode function-unknown proteins. For the seal ST23 strains, 24 genes were found which were present in none of the human ST23 strains, and 21 genes were in the P1 region (Figure 4B, Table 2). The relationships between the genomes were characterized which was based on the cluster of the proteins (presence or absence of a gene in a protein group) by the pangenome matrix. The results visualization in Figure 4A showed that the seal-derived ST23 strains were clustered in one branch, and had the common ancestors with human ST23 strains NNA027, BSE008, NNB011, and NND002 based on their gene content. One of the main differences among the GBS strains was the presence or absence of multiple phage genes inserted in various parts of the genome.

**Table 2:** Gene function of P1 region and coding sequence alignment.

**Gene**	**MRI Z1-201**	**MRI Z1-199**	**FSL S3-026**	**NNA011**	**LZF005**	**515**	**HN016**
	**Function (length)**	P.I.^*^	**P.I**.	**P.I**.	**P.I**.	**P.I**.	**P.I**.
1	hypothetical protein (173)	100	99.42	91.28	0	0	0
2	hypothetical protein (181)	100	99.44	97.22	0	0	0
3	phage encoded DNA polymerase I (657)	100	99.85	91.53	0	0	88.14
4	Phage protein (188)	100	99.47	94.25	0	0	96.55
5	Phage protein (374)	100	99.73	95.43	0	0	91.42
6	hypothetical protein (108)	100	100	94.39	0	0	88.79
7	hypothetical protein (38)	100	100	94.59	0	0	0
8	DNA primase, phage associated (780)	97.82	97.69	93.32	0	0	76.89
9	Phage protein (94)	100	100	91.4	0	0	83.87
10	DNA helicase, phage-associated (459)	100	100	93.67	0	0	91.48
11	unknown phage protein (166)	100	99.39	91.03	0	0	85.28
12	S-adenosylmethionine synthetase (247)	100	99.59	93.71	44.3	44.3	93.01
13	hypothetical protein (49)	100	0	0	0	0	0
14	mRNA interferase YafQ (91)	100	100	0	0	0	0
15	Antitoxin DinJ (binds YafQ toxin) (94)	100	100	0	0	0	0
16	hypothetical protein (152)	100	100	88.08	0	0	86.09
17	Adenine-specific methyltransferase (418)	100	99.04	94.63	0	0	92.94
18	endonuclease of the HNH family (197)	100	100	0	0	0	0
19	DNA-cytosine methyltransferase (393)	100	100	95.66	0	0	94.9
20	hypothetical protein (45)	100	0	0	0	0	0
21	hypothetical protein (84)	100	100	53.01	0	0	0
22	hypothetical protein (130)	97.67	98.41	88.89	0	0	58.59
23	Phage terminase, small subunit (158)	100	99.36	96.82	0	0	96.63
24	Phage terminase, large subunit (521)	100	99.81	98.27	0	20.97	97.5
25	hypothetical protein (98)	100	100	97.94	0	0	0
26	hypothetical protein (113)	100	100	96.43	0	0	0
27	hypothetical protein (68)	100	100	95.52	0	0	0
28	Phage portal protein (446)	100	100	95.67	0	0	97.20
29	Prophage Clp protease-like protein (175)	100	100	78.53	0	49.59	79.31
30	Phage major capsid protein (403)	100	100	89.55	0	0	89.55
31	unknown phage protein (86)	100	100	98.82	0	0	97.65
32	hypothetical protein (113)	100	100	87.5	0	0	91.07
33	prophage pi2 protein 37 (123)	100	98.36	95.08	0	0	95.90
34	prophage pi2 protein 38 (109)	100	100	95.37	0	0	93.52
35	Prophage pi2 protein 39 (190)	100	98.94	98.41	0	0	96.83
36	prophage pi2 protein 40 (140)	99.28	96.40	99.28	0	0	97.12
37	Phage tail length tape-measure protein (1040)	100	97.40	96.82	0	0	96.63
38	hypothetical protein (242)	100	96.68	96.68	0	0	95.44
39	Phage endopeptidase (972)	100	99.04	92.28	0	0	94.75
40	Phage capsid and scaffold (619)	100	99.19	95.47	0	0	95.47
41	Holin (135)	100	96.27	88.06	0	0	71.64
42	Phage-associated cell wall hydrolase (490)	100	95.09	95.5	0	0	61.86
43	Zinc-finger protein (134)	100	95.49	93.98	0	0	0
44	hypothetical protein (54)	100	96.23	96.23	0	0	0
45	Site-specific recombinase (403)	100	98.26	96.27	0	0	39.03
46	Phage integrase (522)	100	99.42	94.24	0	0	48.91

* *Percent identity*.

The sequence alignment results of 11 GBS strains including ST23 human- or seal-derived strains, ST7 fish-derived strain and ST61 cow-derived strain were shown in Figure 4B. Most of the gene coding sequences of these ST23 strains had an over 99% identity, and the region P1 was a common variation region. The P1 region was mainly the prophage sequences with GC content ranged between 42.98 and 43.77% from prophage analysis. The results indicated that compared to human derived strains, the P1 region of seal-derived ST23 strains showed a significantly higher homology with cow-derived ST61 strain (Figure 4B). The detailed results were shown in Table 2. The P1 sequence region from ST7 fish-derived strains showed low homology with human-, seal-, and cow-derived strains. The P1 region consisted 46 coding genes (Table 2). A total 40 genes from the seal-derived ST23 strains had high homology with ST61 cow-derived strain, and 19 gene sequences among them were identical. Genes 13 and 20 were specific for ST23 seal-derived strains and encoded proteins with unknown function. Genes 14, 15, and 18 were specific for seal-derived ST23 strains and cow-derived ST61 strain, which encoded mRNA interferase YafQ, Antitoxin DinJ (binds YafQ toxin), endonuclease of the HNH family, respectively.

## Discussion

Although CC7 and CC552 were the major GBS clones that infect fish, other CC or ST GBS strains were also isolated from fish, such as CC103, ST283 (Delannoy et al., 2013; Godoy et al., 2013). The previous study has indicated that consumption of fish has been associated with an increased risk of GBS colonization in people, and it has been demonstrated that ST283 is a zoonotic GBS clone capable of colonizing and infecting various farmed freshwater fish, causing unusually severe invasive disease in humans (Foxman et al., 2007; Delannoy et al., 2013; Ip et al., 2016; Kalimuddin et al., 2017). Thus, there is a threat to human health for the transmission of GBS in fish. Although ST23 GBS has a broad host range and can infect humans, cattle, dogs, aquatic mammals (seals) and poikilotherms (crocodiles), ST23 GBS has not been identified in fish (Yildirim et al., 2002; Jones et al., 2003; Brochet et al., 2006; Bishop et al., 2007; Delannoy et al., 2013). Infection of tilapia with seal ST23 GBS does not cause the death or symptoms of onset, but the pathogen can be isolated from the kidneys of infected individuals (Delannoy et al., 2016). Three human ST23 isolates from Taiwan area infected tilapia and caused symptoms, but the mortality rate was <30% (Chu et al., 2016). Our study indicated that the mortality rate of tilapia caused by 10 human ST23 isolates was about 70–100%. Histopathological observations also showed that infection of tilapia with human ST23 GBS caused tissue damage and distribution of bacterial cells within the tissue. The different pathogenicity of human ST23 GBS isolates to tilapia may be related to the different virulence of the strain itself. It was also possible that the infection conditions used in different laboratories caused different pathogenicity. For example, an increase in temperature from 28 to 35°C caused near 2-fold mortality in tilapia (Kayansamruaj et al., 2014a).

Ia and III are predominant serotypes of ST23 GBS, of which Ia-type strains predominantly infect human, while type III strains predominantly infect cattle, and serotype Ia GBS is more pathogenic to fish than serotype III (Brochet et al., 2006; Manning et al., 2009; Sorensen et al., 2010; Kayansamruaj et al., 2014b). The phylogenetic analysis showed that the serotypes Ia and III were in different evolutionary branches, and the genetic relationship between Ia strains was very close. CRISPR typing provides deeper discrimination than the current reference method for GBS typing (Lier et al., 2015). The ST23 GBS derived from human, cattle, and seal were in different evolutionary branches, but the CRISPR structure of GBS from seal and human was similar, suggesting that the strains isolated from the seal was likely to originate from human. The complex process of diversity in bacterial population was associated with mutation, transformation, transduction, or conjugation -mediated horizontal DNA transfer. Recombination was a major driver of GBS genetic diversity, which can result in the altered GBS serotype, virulence, as well as the host range (Brochet et al., 2008b; Richards et al., 2011; Da Cunha et al., 2014; Flores et al., 2015; Teatero et al., 2016). The whole genome nucleotide polymorphisms analysis of eight human GBS isolates showed that each chromosome was a mosaic of large chromosomal fragments from different ancestors, indicating that up to 334 kb of large DNA exchanges have contributed to the genome dynamics in the natural population (Brochet et al., 2008b). It has been reported that different CC clones (CC23 and the hypervirulent CC17) can form the new ST (ST452) through large genomic recombination events (Campisi et al., 2016). Prophage DNA fragments are the important insertion sequences associated with GBS horizontal DNA gene transfer, affecting the adaptability and virulence of GBS (van der Mee-Marquet et al., 2006; Domelier et al., 2009). Prophage analysis and genome comparisons showed that prophage consisted of the major difference in the same ST GBS. The seal ST23 GBS, which did not infect the tilapia, had a P1 region consisting of prophage genes, whereas deletion or mutation in P1 region existed in the human ST23 GBS with high virulence to tilapia. In addition, The P1 region from seal ST23 GBS has a higher homology with bovine-specific ST61 GBS strain. Consideration of the report about low virulence of bovine-derived strain to fish (Pereira et al., 2010; Chen et al., 2015), it was speculate that the insertion in the region resulted in the low pathogenicity of seal isolates to tilapia. On the other hands, the deletion or variation of the P1 prophage fragment was the cause of high pathogenicity of human ST23 GBS to tilapia.

The GC contents of the P1 prophage fragment (42.98–43.77%) seriously deviate from the host genomes (35.20–35.70%), suggesting that the prophage was recently acquired and could be specific to other bacterial species. To further elucidate the effect of prophage in the P1 region on the pathogenicity of ST23 GBS to tilapia, we compared the 46 genes encoded in P1 region. Compared to the seal ST23 GBS, three functional genes encoding endonuclease of the HNH family, mRNA interferase YafQ, and Antitoxin DinJ were absent in human ST23 GBS. The endonuclease of the HNH family binds to nucleic acids and possess endonuclease activity, which plays important role in the phage lifecycle as key components of phage DNA packaging machines (Kala et al., 2014). YafQ/DinJ is one of the bacterial toxin–antitoxin (TA) systems (Motiejunaite et al., 2007). TA systems are operons that code for a stable toxic protein and a labile antitoxin, which reduce cell growth to enable the cells to cope with stress (Wang and Wood, 2011). Blast search results showed that the YafQ/DinJ structure was only present in some of the bovine ST61/67 isolates and seal ST23 isolates, and the YafQ/DinJ structure was located in the prophage region, suggesting that GBS could obtain TA systems by phage recombination to improve its adaptability. Therefore, we speculated that seal ST23 isolates obtained YafQ/DinJ structure by phage recombinant, which may improve their growth adaptability and reduce their virulence to tilapia. Further studies are needed to elucidate the effect of phage recombinant or the YafQ/DinJ structure on the pathogenicity of GBS.

## Author contributions

RW, LL, YiH, and TH analyzed data and wrote the manuscript; JT, TX, AL, FL, JL, YaH, YS, and DW performed experiments and analyzed data; MC, QM, and WH conceived and designed the study. All authors reviewed and approved the manuscript.

### Conflict of interest statement

The authors declare that the research was conducted in the absence of any commercial or financial relationships that could be construed as a potential conflict of interest.

## Footnotes

^1^http://rast.nmpdr.org/

^2^http://tree.bio.ed.ac.uk/software/figtree/
