# Corrigendum: Pathogenicity of Human ST23 *Streptococcus agalactiae* to Fish and Genomic Comparison of Pathogenic and Non-pathogenic Isolates

**DOI:** 10.3389/fmicb.2018.00173

**Published:** 2018-02-06

**Authors:** Rui Wang, Liping Li, Yin Huang, Ting Huang, Jiayou Tang, Ting Xie, Aiying Lei, Fuguang Luo, Jian Li, Yan Huang, Yunliang Shi, Dongying Wang, Ming Chen, Qiang Mi, Weiyi Huang

**Affiliations:** ^1^Guangxi Key Laboratory for Aquatic Genetic Breeding and Healthy Aquaculture, Guangxi Institute of Fisheries, Nanning, China; ^2^Institute of Animal Science and Technology, Guangxi University, Nanning, China; ^3^Hechi Center for Animal Disease Control and Prevention, Hechi, China; ^4^Aquatic Animal Disease Pevention and Control Laboratory, Liuzhou's Aquaculture Technology Extending Station, Liuzhou, China; ^5^School of Life Sciences, Fudan University, Shanghai, China; ^6^Guangxi Center for Disease Control and Prevention, Nanning, China; ^7^Aquaculture Laboratory, Guangxi Aquaculture and Animal Husbandry School, Nanning, China

**Keywords:** *Streptococcus agalactiae*, Group B *Streptococcus* (GBS), pathogenicity, genomic comparison, sequence type (ST), ST23

In the published article, there was an error regarding the affiliation for Weiyi Huang. As well as having Affiliation (6), she should also have (2): Institute of Animal Science and Technology, Guangxi University, Nanning, China. The authors apologize for this error and state that this does not change the scientific conclusions of the article in any way.

The original article has been updated.

## Conflict of interest statement

The authors declare that the research was conducted in the absence of any commercial or financial relationships that could be construed as a potential conflict of interest.

