# Supplementary material for: Pathogenicity of Human ST23 Streptococcus agalactiae to Fish and Genomic Comparison of Pathogenic and Non-pathogenic Isolates
Source: Front Microbiol. 2017 Oct 6;8:1933. doi: 10.3389/fmicb.2017.01933 (PMC5635047; doi:10.3389/fmicb.2017.01933)
Supplement: Supplementary file 1 [file Table1.DOC]

**Supplementary Table 1 ST23 GBS strains included in the genomic comparison.**

| **Strain** | **Serotype** | **ST**  **type** | **Host** | **GC content** | **Geographical**  **origin** | **GenBank accession**  **number** |
| --- | --- | --- | --- | --- | --- | --- |
| BSE008 | Ia | ST23 | Human | 35.70% | China | NAWD00000000 |
| LZF005 | Ia | ST23 | Human | 35.20% | China | NAYW00000000 |
| LZF008 | Ia | ST23 | Human | 35.50% | China | NAYU00000000 |
| NNA004 | Ia | ST23 | Human | 35.60% | China | NAYO00000000 |
| NNA011 | Ia | ST23 | Human | 35.70% | China | NAYH00000000 |
| NNA027 | Ia | ST23 | Human | 35.70% | China | NAXL00000000 |
| NNA035 | Ia | ST23 | Human | 35.70% | China | NAXC00000000 |
| NNB011 | Ia | ST23 | Human | 35.60% | China | NAWC00000000 |
| NND002 | Ia | ST23 | Human | 35.70% | China | NAVQ00000000 |
| NND003 | Ia | ST23 | Human | 35.40% | China | NAVP00000000 |
| CCUG 34230 | III | ST23 | Bovine | 35.50% | Unknown | NZ_ANQB01000010 |
| NEM316 | III | ST23 | Human | 35.60% | Unknown | NC_004368.1 |
| 515 | Ia | ST23 | Human | 35.30% | Unknown | NZ_AAJP01000001 |
| MRI Z1-199 | Ia | ST23 | Seal | 35.40% | UK | NZ_ANEH01000024 |
| MRI Z1-200 | Ia | ST23 | Seal | 35.40% | UK | NZ_ANQM01000001 |
| MRI Z1-201 | Ia | ST23 | Seal | 35.40% | UK | NZ_ANQL01000001 |
| MRI Z1-202 | Ia | ST23 | Seal | 35.50% | UK | NZ_ANQK01000001 |
| MRI Z1-203 | Ia | ST23 | Seal | 35.40% | UK | NZ_ANQJ01000001 |
| MRI Z1-204 | Ia | ST23 | Dog | 35.20% | Unknown | NZ_ANQI01000001 |
| GB00867 | Ia | ST23 | Human | 35.20% | Unknown | NZ_ANEH01000024 |
| HN016 | Ia | ST7 | Fish | 35.70% | China | NZ_CP011325 |
| FSL S3-026 | III | ST67 | Bovine | 36.10% | USA | NZ_ANCL01000103 |
| STIR-CD-17 | Ib | ST260 | Fish | 35.30% | Honduras | ALXB01000004 |
